# Optimized Acoustic Phantom Design for Characterizing Body Sound Sensors

**DOI:** 10.3390/s22239086

**Published:** 2022-11-23

**Authors:** Valerie Rennoll, Ian McLane, Mounya Elhilali, James E. West

**Affiliations:** Department of Electrical and Computer Engineering, Johns Hopkins University, Baltimore, MD 21218, USA

**Keywords:** acoustic phantom, sensor characterization, frequency response, stethoscope

## Abstract

Many commercial and prototype devices are available for capturing body sounds that provide important information on the health of the lungs and heart; however, a standardized method to characterize and compare these devices is not agreed upon. Acoustic phantoms are commonly used because they generate repeatable sounds that couple to devices using a material layer that mimics the characteristics of skin. While multiple acoustic phantoms have been presented in literature, it is unclear how design elements, such as the driver type and coupling layer, impact the acoustical characteristics of the phantom and, therefore, the device being measured. Here, a design of experiments approach is used to compare the frequency responses of various phantom constructions. An acoustic phantom that uses a loudspeaker to generate sound and excite a gelatin layer supported by a grid is determined to have a flatter and more uniform frequency response than other possible designs with a sound exciter and plate support. When measured on an optimal acoustic phantom, three devices are shown to have more consistent measurements with added weight and differing positions compared to a non-optimal phantom. Overall, the statistical models developed here provide greater insight into acoustic phantom design for improved device characterization.

## 1. Introduction

Auscultation, or listening to the sounds of the body, provides an accessible, inexpensive, and non-invasive method to monitor the health of the heart, lungs, and intestinal tract. There are a broad range of stethoscopes for auscultation that are commercially available to physicians with different price points, features, and monitoring capabilities from companies such as Littman, American Diagnostic Corporation, Welch Allyn, Eko, Thinklabs, and Sonavi Labs. Recent research has led to stethoscopes with greater resistance to ambient noise corruption [1], onboard algorithmic diagnostic capabilities [2], and lower production costs [3]. New form factors outside of the traditional stethoscope are also developing to target wearable designs that enable continuous body sound monitoring [4,5,6,7,8,9,10]. With such a broad range of existing and developing body sound monitoring technology, it is critical that these devices are accurately characterized and objectively compared to understand their acoustical characteristics and the acoustic signatures they are equipped to detect.

Unlike other sensors that capture physiological data, such as pulse oximeters or electrocardiograms, sound monitoring technology does not have commercially available systems that generate test signals with precise control to characterize and compare the performance of different devices. In publications on developing sensors to capture sound from the body, characterization methods typically use a laboratory acoustic phantom [1,3,4,9], measure on a limited set of human subjects [5,6,7], or use an airborne sound source [9,10]. With such widely varying methods, direct comparisons of the measured characteristics across devices are not robust with dependence on the equipment used or subject population. As body sound monitoring technology can make use of a broad range of sensor technology, including microphones, piezoelectric materials, accelerometers, and capacitive transducers that responds to either changes in air pressure or skin vibrations directly, it is critical that the technology is characterized in a manner that mimics its use case, is comparable across multiple sensors, and excites the device with well-controlled frequency content.

The lack of a complete and standard solution for characterizing body sound monitoring technology is a problem that was identified by Ertel as early as 1966 [11] and has since been explored by multiple researchers. One approach for characterizing body sound monitoring technology is to measure the device on the body of patient [12,13]. While an important characterization step, comparing repeatable sounds with different devices on the same subject can be difficult due to the natural variation of body sounds. To overcome this challenge, Nowak [12] presented a method to extract specific fragments of heart sounds with synchronized electrocardiography measurements to determine the acoustic properties of various stethoscopes on human subjects. While informative, this approach would require a large number of subjects for testing to gain a general characterization of the device’s frequency response regardless of the included subjects anatomical and physiological variations and the specific device placement on the subject.

Due to these challenges, acoustic phantoms that mimic the vibrating conditions of the chest are more commonly used for device characterization because they generate stable and repeatable sounds and offer greater control over the experimental parameters (volume, applied pressure, sound type). Three general acoustic phantom approaches exist, including using a sound source and (1) air coupling [11,14,15], (2) a polymer filled with fluid [16], or (3) a viscoelastic material that mimics skin [17,18,19,20,21,22,23]. The latter approach is more common and offers the opportunity to compare the phantom responses when the viscoelastic material is replaced with real tissue. Researchers have used various designs to combine the sound source and coupling layer that transmits sound to the device under test.

One of the earliest acoustic phantom designs by Kraman [18] used a loudspeaker coupled to a chamber covered by a viscoelastic material and was demonstrated to have similar performance with the viscoelastic material replaced by fat and meat. Zanartu [21] expanded on this design by scaling up the testing platform and using larger loudspeakers that were found to excite low frequencies more effectively. Compared to this design, Mansy [19,20] argued that a sound exciter in direct contact with the coupling material would maximize the amount of energy transferred to the the device under test. The use of a metal plate between the sound exciter and coupling layer was also found to improve spatial uniformity across the phantom surface. Klum [17] also used a structure-borne sound converter and found that the acoustical characteristics of the phantom changed with temperature, coupling layer castings, and added weight. Nelson [23] followed a design similar to Mansy and Klum, but introduced a shaker in the setup to generate ambient vibration signals that would simulate those found in helicopters. In a change from other designs using sound exciters, Weiss [22] made use of a plastic grid and castor oil; however, the authors were only interested in comparing devices, so the phantom was not characterized in detail.

Comparing these acoustic phantoms, it is evident the design choices are critical and impact the system’s acoustical characteristics and stability over time; however, no clear evidence is presented that supports one phantom design over another. Many ambiguities remain over the benefits of using a sound exciter compared to a loudspeaker, how different phantom designs respond to added weight, and when equalization processing is necessary to whiten the acoustic phantom response to characterize a device. Moreover, existing literature lacks sufficient detail that a person not familiar with the work could easily replicate the design and characterization process due to the use of inaccessible processing code and nonspecific construction steps. The properties of acoustic phantoms used to measure body sound sensors are critical as they can shape the characterization of the device under test.

To expand on previous publications, this study aims to propose a standardized phantom design and characterization technique. An acoustic phantom was constructed with a modular design that allowed the driver, coupling layer, and support layer to be easily exchanged. Following a design of experiments (DOE) approach, the frequency responses of 35 phantoms with variations in the base design and several added weights were measured using a laser vibrometer. Quantitative metrics were extracted from the measured frequency responses and used to develop statistical models that inform how the phantom can be designed to demonstrate a more flat, uniform, and robust response with added weight. Similar models were also developed to understand how to minimize the phantom’s generation of airborne noise and susceptibility to vibrations from surrounding airborne noise.

Using the developed models, an optimal phantom design was chosen and measured for various robustness considerations, including its stability over time, changes with applied weight, and behavior with real tissue instead of gelatin. The frequency responses of several devices, including two electronic stethoscopes and a piezoelectric material, were measured using an optimized and non-optimized acoustic phantom. For cases where it is desired to alter the phantom frequency response further, a whitening process is presented that minimizes the remaining peaks and dips in the frequency response. We aim to present the acoustic phantom design and characterization with explicit details that can be easily followed by researchers developing new body sound monitoring technology and in need of methods to objectively compare devices.

## 2. Methods

An acoustic phantom with a modular design is presented along with characterization methods to measure the frequency response, airborne noise production, and susceptibility to airborne noise of various constructions. Quantitative metrics are extracted from the measured acoustic responses and used to develop statistical models that inform an optimal acoustic phantom design, which is further characterized for robustness considerations and with a whitening procedure.

### 2.1. Phantom Construction

**Figure 1:**
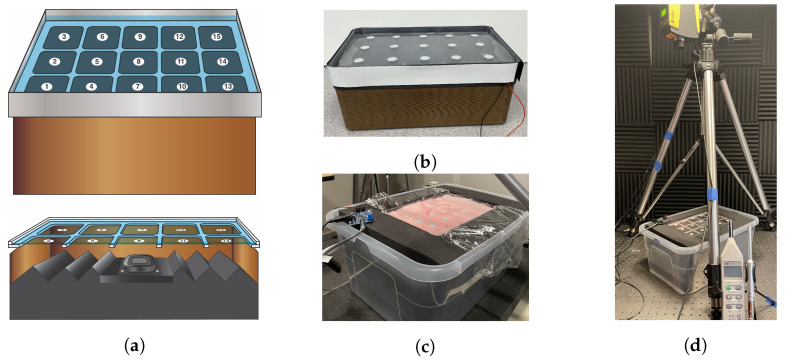
(**a**) The acoustic phantom design with the grid layer and construction with (**b**) a gelatin or (**c**) a pig skin coupling layer. (**d**) The characterization setup for the acoustic phantom with the laser vibrometer on a tripod pointing towards a reflective tape position.

An acoustic phantom requires two main components, a sound source and a layer that mimics human tissue to couple to the device under test. In addition to incorporating these two elements, the phantom design and construction was shaped by preferences for low-cost construction, a modular design that allowed components to be easily exchanged, and a surface area large enough to hold multiple devices. The base design of the acoustic phantom, shown in Figure 1, was constructed using a 9 inch × 6 inch × 3.25 inch polypropylene box (2020 Target Brands) and a 9 inch × 6 inch × 0.75 inch stackable lid (2020 Target Brands) that held a gelatin layer. The lid fit tightly within the box and was secured further with magnetic tape around the seam between the two components. Several layers of acoustic foam were stacked within the box to prevent resonances. On the surface of the gelatin, a stencil is used to place reflective tape at 15 positions across the phantom surface where the vibration is measured. Positions 5, 8 and 11, shown in Figure 1a, will be referred to as left center, center, and right center throughout the paper. The phantom is placed within a larger box and press-fit firmly within foam. The foam holds the phantom in place and also minimizes sound propagation from the sides of the phantom.

Within this basic design, five factors, shown in Table 1, were varied to understand their impact on the measured frequency response characteristics of the phantom. The driver was either a loudspeaker (HiWave BMR12 Compact 2” Full-range speaker, HiWave, Springboro, OH, USA) that produces airborne sound to vibrate the gelatin layer or a sound exciter (Dayton Audio DAEX25, Dayton Audio, Springboro, OH, USA) that directly vibrates the lid or gelatin layer. The loudspeaker is reported by the manufacturer to have a frequency response between 150 and 16,000 Hz. The sound exciter is reported to have a wideband frequency response by the manufacturer, but the specific frequency response is dependent on the attached diaphragm material, the sound exciter placement on the diaphragm, and how the sound exciter is loaded. Both drivers were found to excite the full frequency range of interest (50 to 8000 Hz) when used in the acoustic phantom and measured by a vibrometer. The driver of choice was connected to an amplifier (Adafruit MAX9744 Stereo 20W Class D, Adafruit, New York, NY, USA) with an adjustable gain. The loudspeaker was held tightly in stiff foam at the center of the box and the sound exciter was adhered directly beneath the center of the lid. The lid, which acts as the gelatin support layer, comes in two forms-either the solid lid (‘plate’) or the lid with 15, laser-cut 4 cm × 4 cm squares (‘grid’). The square cutouts were sized to roughly match the diameter of stethoscope diaphragms.

The gelatin layer placed on the lid is made of varying types and thicknesses of gelatin. The gelatin, purchased from Humimic Medical, is synthetic, stable at room temperature, and can be purchased with various material and acoustic properties. Gelatin types two, three, and five were used due to their range of acoustic impedance values (1.41, 1.25, and 1.64 MRayls, respectively). To form the gelatin layer, the gelatin is melted in an oven around 100 °C, poured into a baking sheet, and, when cured, cut to match the lid size. The gelatin thicknesses varied approximately between 2, 6, and 10 mm. The exact thickness was measured using calipers at ten randomly chosen positions around the gelatin edge and averaged. To understand the effect of applied weight, calibrated weights with slots (200 g or 500 g) are placed at the center position of the phantom. The weights have an approximately 10 cm hole that allows for vibrometer measurements to be collected at the center of the applied weight. Considering the applied weight is important because any device will load the phantom to some degree and additional weight is often applied to guarantee the device makes full contact with the gelatin layer and to simulate a physician pressing the stethoscope on a patient. More detailed information, including images of the phantom components and its construction protocol, is provided in the Supplemental Material.

### 2.2. Characterization

The constructed phantoms were excited with a sine sweep and the resulting vibration was measured using a laser vibrometer, as shown in Figure 1d, to characterize the frequency response at 15 positions marked by a stencil. The airborne noise generated by the phantom and the phantom’s response to surrounding airborne noise were also measured. All measurements were collected in a sound isolation booth and the phantom was supported by several layers of acoustic foam for additional isolation from noise conducted through the ground or support table.

#### 2.2.1. Frequency Response

To determine the frequency response of the phantom, the driver was excited with a sine sweep and the resulting vibration at the gelatin layer was measured using a laser vibrometer (Polytec PDV-100, Polytec, Hudson, MA, USA). The laser vibrometer was set to a velocity measurement range of 20 mm/s, which provides the highest resolution. Room EQ wizard (REW) [24], room acoustics software, generated the sine sweep and analyzed the phantom vibration captured by the vibrometer using the methods outlined by Müller [25]. REW is an application for measuring room responses and countering room modal resonances. Among other capabilities, the software includes tools for generating test signals, and measuring frequency and impulse responses. While originally intended for room acoustics measurements, the software’s capabilities translate well to measuring and countering resonances in an acoustic phantom’s response as well. The software was used for characterization because it is easily accessible, well-documented, and straightforward to use.

The software sends a logarithmically swept sine signal to the source of interest (here, the phantom driver) and then the resulting response can be measured using a vibrometer, accelerometer, or microphone. A vibrometer was used because it is a non-contact method that is insensitive to ambient noise. Unlike a microphone, the vibrometer measures vibration at specific points rather than averaging over a larger surface. The sine sweep conditions were set to a frequency range between 20 and 10,000 Hz, a length of 1 Ms, a −12 dBFS RMS signal level, a 44.1 kHz sample rate, and a single repetition. The sine sweep generated by REW was sent from the computer to the phantom’s driver amplifier. The vibrometer output was sent through an audio interface (Focusrite Scarlett 2i2) and recorded from the computer. The computer volume and amplifier gain were set such that the vibrometer reading was approximately −45 dBFS at the center position. This level was chosen as it was the lowest reading that applied across all acoustic phantom designs. The audio interface gain was then adjusted until the signal level was approximately −18 dBFS to balance added noise that occurs at lower levels and clipping that occurs at higher levels.

Reflective tape with a diameter of 1.5 cm was placed at 15 positions (2 cm distance between each position) across the surface of the acoustic phantom and used as the measuring points for the laser vibrometer. Each of the 15 positions was measured one time, then the gelatin was removed and readjusted on the support layer, and this process was subsequently repeated for a total of three times, leading to 45 frequency response measurements per phantom (15 positions times 3 trials). The response for each position is reported as the root mean square average in the linear scale across the three repeated measurements to account for variability due to slight differences in the vibrometer placement. The vibrometer and audio interface were measured and found to have flat frequency responses in the range of interest that would not interfere with the acoustic phantom frequency response.

#### 2.2.2. Airborne Noise

While the acoustic phantom’s frequency response was of primary interest, we also aimed to understand the relationships between the design factors and (1) the amount of airborne noise the acoustic phantom produces and (2) how the phantom responds to surrounding airborne noise. These characteristics are important for experiments quantifying how airborne noise interferes with a device capturing the signal of interest, as found in [1,20]. Ideally, the acoustic phantom would generate less airborne sound comparable to that of actual body sounds and would be less susceptible to vibration with airborne noise so that the noise does not travel through the gelatin layer to excite the device under test.

To measure the airborne noise generation and susceptibility, four noise types were chosen, including 10 s long clips of pink noise, white noise, music (Beethoven Symphony No. 5), and speech (’clnsp0.wav’ from the Microsoft Scalable Noisy Speech Dataset). To measure how susceptible the phantom was to airborne noise, the vibrometer was pointed at the surface of gelatin and a signal was recorded in silence (b(t)) and while the four noise types ($n_{speakers}\left(t\right)$) were played from two surrounding speakers (Yamaha HS8) at an average loudness of 80 dB. An SNR (SNR${}_{susceptibility}$) was calculated as:

(1) $$\begin{array}{c} {\mathrm{SNR}}_{susceptibility}=\frac{\sqrt{\sum n_{speakers}{\left(t\right)}^{2}}}{\sqrt{\sum b{\left(t\right)}^{2}}} \end{array}$$

A lower SNR${}_{susceptibility}$ indicates that the acoustic phantom is less susceptible to airborne noise.

To measure the airborne noise generation of the phantom, a calibrated microphone (Dayton Audio EMM-6), placed approximately 10 cm from the phantom, recorded a baseline signal (b(t)) in silence followed by recording the four noise types ($n_{phantom}\left(t\right)$) played from the phantom at a consistent volume. An SNR (SNR${}_{generation}$) was calculated as:

(2) $$\begin{array}{c} {\mathrm{SNR}}_{gen}=\frac{\sqrt{\sum n_{phantom}{\left(t\right)}^{2}}}{\sqrt{\sum b{\left(t\right)}^{2}}} \end{array}$$

A lower SNR${}_{generation}$ indicates that the acoustic phantom generates less airborne noise. Both SNR${}_{susceptibility}$ and SNR${}_{generation}$ were averaged in the linear scale across 15 trials and the 4 noise types measured.

### 2.3. Design of Experiments

DOE is a statistical approach for experimental planning that allows for a design space to be systematically and efficiently explored to understand the factors that have a significant impact on a response [26]. Compared to one factor at a time experimentation, which measures the change in the response when a single variable is changed and all others are held constant, most DOE approaches vary factors simultaneously so that interactions between factors can be explored. When following a designed experiment where the factors are varied independently, a model can be developed that links the factors and measured response. While there are many approaches to DOE, optimal designs are a general purpose tool to obtain statistically grounded designs in the presence of nonstandard experimental conditions [27]. Optimal design of experiments offer flexibility in tailoring the design to the boundaries of the problem at hand, rather than tailoring the experiment to fit a classical design with specific restrictions. Here, an I-optimal design was generated in JMP [28] to determine the relationship between the varied factors, shown in Table 1, and responses relevant to the acoustical characteristics of the measured phantoms. Compared to existing research on acoustic phantom designs, the design of experiments approach used here provides statistical evidence for choosing specific acoustic phantom design elements and allows for interactions between design factors to be understood.

#### 2.3.1. Treatments

In total, 35 phantom designs were measured with varying gelatin types, thicknesses and supports, drivers, and applied weights. The treatments, shown in Table 2, were determined using JMP and selected to have reasonable power to detect all significant main effects, second order interactions, and quadratic terms.

#### 2.3.2. Responses

To create a model linking the phantom design factors and the corresponding acoustical characteristics, the measured data was summarized into six continuous response values. The chosen values were extracted as a proxy to describe the flatness and uniformity of the measured frequency response and the susceptibility to and generation of airborne noise. As a large number of frequency responses (45) were collected for each simulator, visual comparisons would be difficult and not allow for easily understandable statistical inferences.

The flatness of the frequency response was summarized using the slope and R-squared values extracted from a linear regression line fitted to the average frequency response of each phantom measured at the center position. Ideally, a slope closer to zero and an R-squared value closer to one would indicate that the frequency response is fairly flat with few departures from the fitted line. The uniformity of the frequency response across the 15 positions of the phantom surface was then determined with two additional values by calculating the average Euclidean distance and correlation between the frequency responses at the center position and all other positions. An acoustic phantom with a more uniform frequency response across its surface would have a smaller Euclidean distance and correlation closer to one, indicating that both the shape and magnitude of the frequency responses across the surface are similar. To quantify the amount of airborne noise generated by an acoustic phantom and how much vibration is induced in the phantom due to surrounding airborne noise, SNR${}_{generation}$ and SNR${}_{susceptibility}$ were used, as described in Section 2.2.2.

#### 2.3.3. Model Fitting

Six models between the factors and each of the six responses were determined using standard least squares in JMP. Non-significant factors, typically with the significance level set to 0.05 or 0.01, were removed using backwards elimination while maintaining model hierarchy. The significance level was set to 0.05, unless the R-squared value calculated via 5-fold cross-validation increased or decreased by less than 0.05 at a significance level of 0.01, in which case a significance level of 0.01 was used. The goal was to explain the variation in the response with as few terms as possible and confirm that model diagnostics were met.

The models were diagnosed visually and with statistical tests to confirm that no outlying or influential points skewed the model (assessed using leverage and Cook’s distance), the errors were normally and independently distributed with a mean of zero and constant variance (assessed using the Shapiro–Wilk test), and the model demonstrated no significant lack of fit. The linear regression models were combined using the prediction profiler feature in JMP. The prediction profiler optimizes a desirability function to determine what factor settings target specific response values. Four features were targeted, including (1) the flattest frequency response at the center position (indicated by a fitted line with a slope near 0 and high R-squared value), (2) the most uniform response across the surface (indicated by a low Euclidean distance and high correlation across positions), (3) the least susceptibility to ambient noise (indicated by low SNR${}_{susceptibility}$), and (4) the least generation of airborne noise (indicated by low SNR${}_{generation}$).

### 2.4. Optimal Phantom Testing

Using the prediction profiler, an acoustic phantom design was selected that showed the most optimal characteristics (flattest response at the center position with highest uniformity across the surface and lower generation of and susceptibility to airborne noise) and fully characterized to determine how robust the phantom was to small changes in the testing conditions and over time. The average correlation and root mean square error (RMSE) between the frequency responses of the baseline phantom and the same phantom with changes in the gelatin layer, applied weight, or excitation volume were compared across the 15 positions. For comparison purposes, the average frequency responses were all shifted around their mean. The baseline acoustic phantom was compared to the same simulator with:

1. the coupling layer replaced by a layer of 4.7 mm pig skin supported by a thin layer of plastic wrap for hygienic purposes,
2. the same gelatin layer allowed to sit for one month undisturbed,
3. the same gelatin layer melted and reformed,
4. a new gelatin layer (same type and approximate thickness) measured with 90 and 300 min allowed for curing,
5. three new gelatin layers of the same type and approximate thicknesses,
6. added weights of 50, 200, and 500 g applied at the center position,
7. two 200 g weights placed at the left center and right center positions,
8. a 500 g weight at the center position applied for 2 full days, and
9. with a lower and higher excitation volume that test the upper and lower limits of the system.

### 2.5. Device Measurements

To demonstrate the use of the acoustic phantom to characterize body sound monitoring technology, three devices were measured, including the Thinklabs One (using the center setting), the Sonavi Labs Feelix digital stethoscope, and a piezoelectric PZT disk. Each device was measured on an optimized and non-optimized phantom and excited with a sine sweep at the center position with 0 and 200 g of added weight and at the left center position with no added weight. The devices were measured three times for each condition and the responses were averaged. The optimized simulator used a loudspeaker and grid supporting 6.5 mm of gelatin type two. The non-optimized simulator used a sound exciter and grid supporting the same gelatin layer. For device testing, a slightly thicker gelatin was used as it was found to provide greater support to the devices under test. The simulator output volume was adjusted to a uniform level for each phantom using the laser vibrometer and then the input gain for each device was adjusted to an appropriate level to capture a clean signal. The goal of these measurements was to demonstrate (1) the use of the acoustic phantom for characterizing devices that measure either an acoustic or vibrational response, (2) that device measurements with an acoustic phantom allow for objective comparisons, and (3) that the optimized acoustic phantom provides more uniform device measurements across its surface and with added weight.

### 2.6. Whitening

The goal of this study is to design a phantom with as flat and uniform frequency response as possible; however, there are certain instances where equalization might be required to further mitigate frequency peaks and valleys that remain regardless of the phantom construction. Using REW, a filter is calculated to fit the measured frequency response that should be mitigated. The filter settings are exported from REW and imported to Equalizer APO [29], which applies the inverse filter to the output sound from the computer that is sent to the acoustic phantom.

To demonstrate this whitening procedure, a filter is computed and applied using the average response measured from the center position of an acoustic phantom with approximately 5 mm gelatin (type 2) supported by a grid and excited with a loudspeaker. In REW, the settings were applied to target the frequency response with an overall flatness target of 1 dB in the range of 50 to 3000 Hz. Other settings provided by REW were determined empirically to optimize the fit between the measured frequency response and calculated filter. The frequency response of the phantom was remeasured at the center position with and without equalization applied to demonstrate its effect.

## 3. Results

### 3.1. Frequency Responses

**Figure 2:**
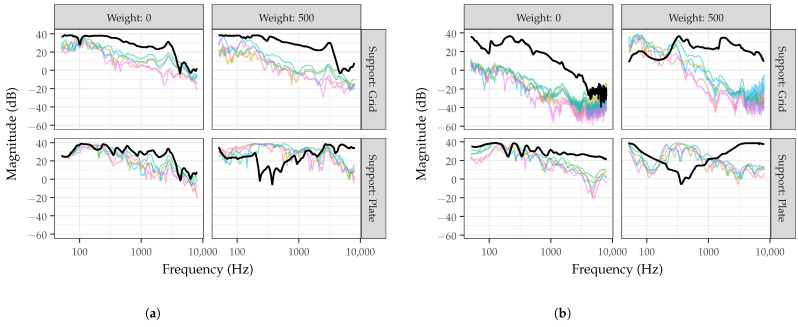
Frequency responses measured from phantom designs with (**a**) a loudspeaker or (**b**) a sound exciter and tested with 0 and 500 g added weight while the gelatin is supported using either a grid or plate. The black line shows the frequency response measured from the center position, while the colored lines indicate frequency responses measured from one of the other 14 positions.

Figure 2 shows measured frequency responses from eight phantoms using either a loudspeaker (Figure 2a) or sound exciter (Figure 2b) with 0 or 500 g of added weight and the gelatin supported by either a grid (top row) or plate (bottom row). The phantoms used various gelatin types, but were all approximately 2 to 4 mm thick. The thick, black lines show the frequency responses measured from the center position, while the thin, colored lines show the frequency responses measured from the 14 other positions. While the figure only includes a small subset of the measured phantoms for illustration purposes, there are several trends that indicate the phantom design parameters have a significant impact on the frequency response.

Considering the frequency responses measured from the center positions, no phantoms demonstrate a flat response across all frequencies, but instead have various peaks and valleys. Between approximately 100 and 3000 Hz, the loudspeaker with grid support demonstrates the smoothest frequency response with a fairly linear decrease in magnitude and minimal peaks and valleys. Above 3000 Hz, both phantoms using a loudspeaker without added weight show a fairly steep drop in magnitude. Comparatively, the phantom using a sound exciter and plate without added weight shows a more linear drop off across the entire frequency range, but with a greater number of peaks and valleys. Without added weight, the sound exciter with grid support demonstrates a steeper drop off in magnitude with increasing frequency compared to the plate support.

**Figure 3:**
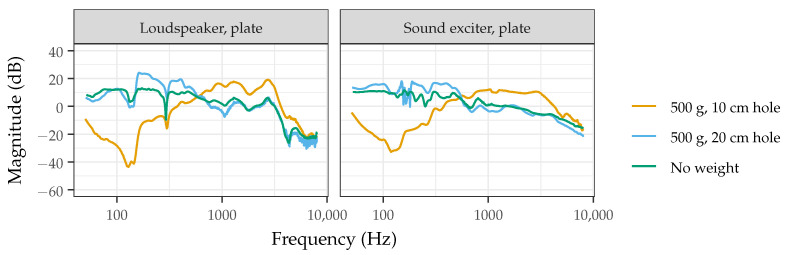
Frequency response measured from the center position of the acoustic phantom without added weight or with 500 g of added weight supported by an acrylic disk with either a 10 cm or 20 cm center cutout.

With added weight, the phantom using a loudspeaker and grid support visually appears to be more robust. At the center position, this phantom design has a similar frequency response with and without 500 g of added weight, compared to other designs using a sound exciter or loudspeaker with plate support. In particular, phantoms using a plate for gelatin support with 500 g of added weight show a prominent dip near 200 Hz. This frequency dip was hypothesized to be present for phantoms using a plate support with added weight for several possible reasons, including the position of the driver or weight, or the amount of weight applied. Upon further investigation, it was determined that this frequency dip was present regardless of these factors and instead was a result of the area that was allowed to freely vibrate beneath the calibrated weight. The weight was placed on an acrylic support with a 50 cm diameter and a circle cutout with varying diameters (10, 20, 30, or 40 cm). As shown in Figure 3, when the diameter of the cutout increased from 10 to 20 cm, the frequency dip was no longer present and the frequency response approached that of the phantom without added weight. While more research would be necessary to elucidate this response, it is hypothesized that the applied weight with the center cutout changes the boundary conditions of the vibrating gelatin in this small area and that care should be made in the weight distribution of devices when placed on the phantom.

Comparing the center (black line) and outer positions (colored lines) in Figure 2, it is noted that the frequency response varies across the surface of all simulators to varying degrees. In particular, with added weight, the sound exciter and loudspeaker with plate support demonstrate that the center position has a significantly different frequency response than the other positions. Without added weight, the sound exciter with a grid support has a significant decrease in magnitude at positions away from the center. Overall, the visual comparison of frequency responses for acoustic phantoms with 3 mm thick gelatin indicates that a loudspeaker with a grid support is the flattest, most uniform, and most robust to added weight.

### 3.2. Design Considerations

**Figure 4:**
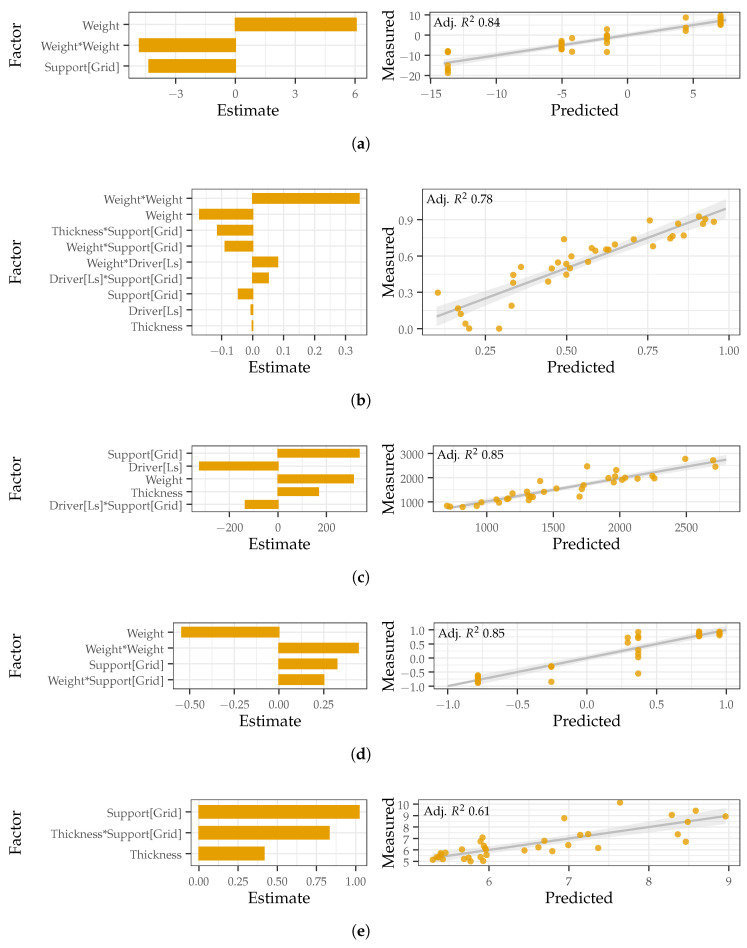
The significant factor estimates (**left**) and predicted versus measured plots (**right**) for the (**a**) center position slope, (**b**) center position R-squared, (**c**) Euclidean distance, (**d**) correlation, and (**e**) SNR${}_{susceptibility}$.

Compared to visual frequency response comparisons, the DOE approach with linear regression allows for specific relationships between the phantom factors and responses of interest to be explored in greater detail. The responses (slope and R-squared values for linear regression line fitted to center position frequency response, average Euclidean distance and correlation between the frequency response at the center position and all other positions, SNR${}_{generation}$, and SNR${}_{susceptibility}$) are used as proxies to describe the measured acoustical phantom characteristics, including the flatness and uniformity of the frequency response and the susceptibility to and generation of airborne noise. Significant relationships were identified between the investigated factors and all responses, except for SNR${}_{generation}$. Figure 4 shows the significant factor estimates and predicted versus measured line for each of the five responses that were modeled. A larger factor estimate indicates that a factor has a greater impact on the response, with positive estimates increasing the response and negative estimates decreasing the response. For categorical factors, such as the gelatin support, the estimate is shown for one factor level. Another factor level will have the same estimate, but of opposite sign. The predicted versus measured line indicates how well the measured data fits with the values predicted by the model. When the measured data points are close to the line of best, this indicates a better fit. Overall, the factor estimates indicate that all of the factors studied, except for the gelatin type, are significant to at least one of the responses studied. In particular, the applied weight and support type are the most significant factors for 3 and 2 of the responses, respectively. In addition to significant main factors, each of the response models also includes significant quadratic or interaction terms. The models all had a fairly decent fit with adjusted R-squared values above 0.6.

To indicate the overall flatness at the center position, the slope and R-squared value of a line-of-best fit were used. The models for slope and R-squared fit well with adjusted R-squared values of 0.84 and 0.78, respectively. Significant factors for the slope include the applied weight, a quadratic of applied weight, and the gelatin support. The slope is maximized with added weight and a plate support, but minimized with no added weight and a grid support. To target a slope near 0, or a more flat frequency response at the center position, the model indicates a grid with 500 g of applied weight is optimal. Significant factors for the R-squared value included four main effects (weight, gelatin support, driver type, and gelatin thickness), a quadratic term (applied weight) and four interactions (gelatin thickness and support, applied weight and gelatin support, applied weight and driver type, and driver type and gelatin support). To target a high R-squared value, or frequency response with fewer departures from the line of best fit, a phantom with a sound exciter and plate supporting thicker gelatin and no applied weight is optimal. Combining the models for slope and R-squared, the results indicate that to target a flat response with and without added weight at the center position, the best phantom designs include a sound exciter with a plate supporting 12 mm thick gelatin or a loudspeaker with a grid supporting 2 mm thick gelatin, respectively.

To indicate the uniformity of the phantom frequency response across the surface, the average Euclidean distance and correlation were used. The models for Euclidean distance and correlation fit well with adjusted R-squared values of 0.85 and 0.85, respectively. Significant factors for the Euclidean distance include the gelatin support, driver type, applied weight, gelatin thickness, and an interaction between the driver type and gelatin support. To target a low Euclidean distance, or minimize magnitude differences in the frequency response across the surface, a phantom with a loudspeaker and plate supporting thinner gelatin and no applied weight is optimal. Significant factors for the correlation include the applied weight and its quadratic, the gelatin support, and an interaction between the applied weight and gelatin support. To target a high correlation, or better match the frequency responses across positions, a phantom with no applied weight and a grid support is optimal. Combining the models for Euclidean distance and correlation, to target a uniform response with and without added weight, the optimal phantom design uses a loudspeaker with a plate supporting 2 mm gelatin or a loudspeaker with grid supporting 2 mm gelatin.

To indicate the susceptibility to airborne noise, SNR${}_{susceptibility}$ was used. The model had an adjusted R-squared value of 0.61 and the gelatin support, thickness, and an interaction between the two were significant factors. A thicker gelatin with a plate support is less susceptible to airborne noise. To indicate the generation of airborne noise, SNR${}_{generation}$ was used. After removing non-significant terms (*p*-value < 0.01), the model was found to have a poor fit with an adjusted R-squared value of 0.28. This indicates that the factors included are not able to explain the variation measured in the SNR value and it is likely that no significant difference was present between the phantom designs and the amount of airborne noise that was generated. As such, this response model is not included in Figure 4.

To interpret the response models as a whole, a target level for each response is set and the desirability is maximized across all models. An acoustic phantom with added weight and a loudspeaker exciting a grid supporting 2 mm gelatin is determined to be the flattest, most uniform, and least susceptible to airborne noise.

### 3.3. Optimum Phantom

The phantom used a loudspeaker with a grid supporting an approximately 3 mm thick, type 2 gelatin. Negligible changes in the responses were impacted by the gelatin type, so gelatin 2 was selected as its acoustic impedance is closest to that of skin. Table 3 shows the correlation and RMSE measured between a baseline, optimal phantom and the same phantom with a parameter changed to understand its similarity to real tissue, stability over time or with changes in the gelatin layer, and robustness to added weight or different excitation volumes. Overall, the acoustic phantom was found to be fairly robust to all parameters; the lowest correlation and highest RMSE values were 0.91 and 4.12, respectively, for comparing a phantom with and without 500 g of added weight. The high correlation values above 0.9 in all testing conditions indicate that: (1) the acoustic phantom behaves similarly with gelatin and real tissue, (2) the phantom behavior is stable over time, (3) the gelatin layer can be reformed and a similar response will be obtained, (4) the phantom exhibits no significant changes after allowing curing for 90 min, (5) similar results are obtained with new gelatin layers that have slight variations in thickness, (6) the phantom is robust to added weight at different positions up to 500 g, and (7) the frequency response is independent of the excitation volume.

### 3.4. Device Measurements

**Figure 5:**
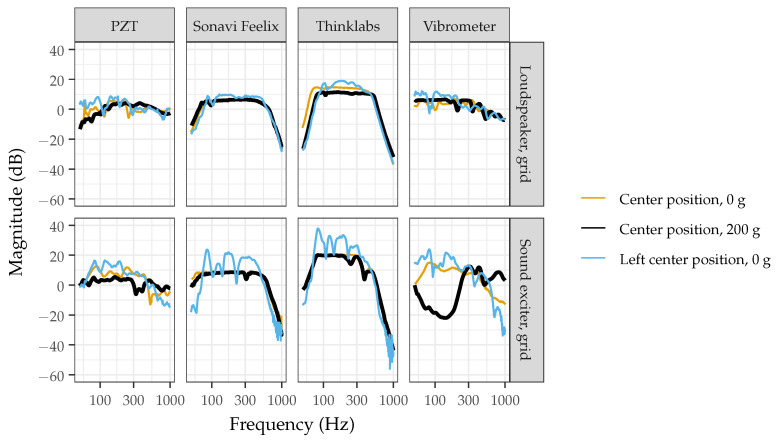
Frequency responses measured from a piezoelectric PZT disk, two digital stethoscopes (Feelix, Thinklabs), and vibrometer on an optimized (loudspeaker with grid support for 6 mm, type 2 gelatin) and non-optimized (sound exciter with grid support for 6 mm, type 2 gelatin) acoustic phantoms. The frequency responses are shown when measured at the center position with 0 and 200 g of added weight and at the left center position with no added weight.

Figure 5 shows the frequency responses measured from several devices on an optimized and non-optimized acoustic phantom. The frequency responses were measured at the center position with 0 and 200 g of added weight and at the left center position with 0 g of added weight. Compared to the non-optimized acoustic phantom, the optimized acoustic phantom provides frequency response measurements that are more comparable with added weight and across positions. When measured at the left center position, the non-optimized simulator shows several high magnitude peaks and valley. When measured on the optimal phantom, the frequency response of the Thinklabs One is more comparable to the specifications listed by the manufacturer—a flat frequency response between 80 and 500 Hz [30]. With and without added weight on the non-optimized phantom, the Thinklabs has valleys at 225 and 380 Hz. For the optimized phantom, the least repeatable response is for the PZT disk with and without added weight. The piezoelectric material was expected to behave differently with added weight, so this is likely a contribution from the material itself and not a factor of the acoustic phantom.

### 3.5. Whitening

**Figure 6:**
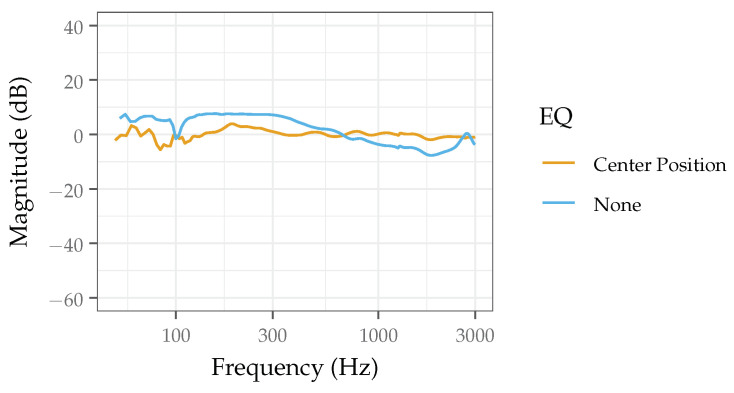
Frequency response measured from the center position of the acoustic phantom with and without equalization applied.

Figure 6 shows the frequency response of the phantom measured at the center position with and without equalization applied. The equalization process did improve the overall flatness of the spectrum; with and without equalization, the frequency response spanned approximately 5 and 7 dB from the average magnitude, respectively. The whitening particularly improved the flatness of the frequency response above 300 Hz, where the span is below 3 dB. However, imperfections still remain below 300 Hz, which could be further mitigated by applying multiple filters via an iterative whitening procedure. Errors in the whitening procedure would be expected due to (1) imperfections in the filter calculation or (2) the acoustic phantom exhibiting nonlinear behavior that equalization cannot account for. Therefore, while whitening is a useful tool and does help to improve the flatness of the frequency response, it is not a perfect process, which is why the phantom design should first be optimized to reduce the need for a whitening procedure. While the whitening process has been demonstrated here, the specific procedure (filter(s) used) followed will vary based on the experimental goals.

## 4. Discussion

The results presented provide a thorough and detailed overview of the acoustical characteristics of acoustic phantoms with various designs and lead to several relevant findings: (1) the chosen phantom design factors have a significant effect on the measured frequency responses, (2) certain phantom designs have a flatter and more uniform frequency response with greater robustness against added weight, (3) the optimal phantom, which uses a loudspeaker and grid support, is robust over time, behaves similarly to real tissue, and more reliably measures devices, and (4) equalization can minimize remaining dips and bumps if necessary. Overall, the study provides a complete overview of considerations for a researcher considering constructing an acoustic phantom to characterize body sound monitoring technology.

Compared to previous literature on acoustic phantom designs, the approach here determined an optimal acoustic phantom design with supporting evidence from statistical models that show the effect of phantom design components on measured acoustical characteristics. Similar to [19], the results indicate that a plate support for the gelatin layer can help improve uniformity in certain cases, but we demonstrate there are limitations when additional weight is applied. Compared to [17], the optimal acoustic phantom is shown to be robust to added weight up to 500 g and stable beyond 60 h of use. In [22], the Thinklabs stethoscope was also measured for objective comparisons to other stethoscope devices using a phantom. Compared to the reported flat frequency response characteristics by the manufacturer, [22] found a dip in the frequency response around 250 Hz, which highlights the need for an improved phantom design. While a comprehensive acoustic phantom study that expanded upon the results presented in past literature, this was not an exhaustive study of all possible factors that could be significant to the frequency response of an acoustic phantom. It is likely that there are still limitations that could be expanded upon, such as further studying weight distribution, determining how these results vary with the size of the phantom enclosure and drivers used, and measuring the full phantom surface rather than specific points.

To expand on the results shown in this study, a follow-up study would be helpful to determine how replicable the measured phantom frequency response is when the phantom is constructed with researchers from different institutions. Researchers would be given the necessary supplies and protocols to construct and characterize the acoustic phantom. The frequency responses measured from different researchers constructing the same phantom could be compared to understand if the phantom could be given a single frequency response, such that any researcher could construct the phantom and not need a laser vibrometer, a fairly expensive piece of equipment, for characterization. Additionally, the acoustic phantom presented here makes use of unnatural sounds, the sine sweep, to characterize the device under test. This is critical to guarantee that the device is excited with all possible frequencies of interest, however, such a sound would be unnatural for the body to generate in practice. As middle ground between characterizing the device on an acoustic phantom with an artificial sound source and characterizing on a real subject, it would be helpful to modify the acoustic phantom to use a preserved lung or heart to generate the sound.

## 5. Conclusions

Developments in audio signal processing, flexible materials, and wearable sensors are leading to a range of new body sound monitoring technology. Prior to characterizing these devices on real subjects, it is critical to demonstrate these devices have the necessary sensitivity and frequency response to capture body sounds and acoustic signatures that indicate health conditions. Although multiple characterization approaches for body sound monitoring technology have been presented, a standard method is not agreed upon. Acoustic phantoms are most often used, but minimal research has been done to thoroughly investigate design components of acoustic phantoms. The presented work addresses the need for more robust body sound monitoring characterization methods by comparing the acoustical characteristics of multiple acoustic phantom designs. Through the generation of a statistical model, it is determined that phantoms using a loudspeaker and grid to support the coupling layer allow for a more flat and uniform frequency response across the surface without any equalization processing. The construction of the acoustic phantom is presented with specific details and its characterization uses freely available software so that other researchers can easily follow this approach for characterizing body sound monitoring technology. The critical study of acoustic phantom design considerations will provide a means for researchers to better characterize and compare developing body sound monitoring technology. Rather than using a loudspeaker, future work will investigate the use of a preserved lung or heart for sound generation, which will allow the phantom to more closely match the conditions found when testing devices on a human subject.

## Figures and Tables

**Table 1:** The five factors of the phantom construction varied in the design of experiments to understand their impact on the measured frequency response characteristics.

Factor	Type	Levels
Gelatin thickness	Continuous	2, 6, 10 mm
Gelatin type	Categorical	2, 3, 5
Driver type	Categorical	Speaker, Exciter
Gelatin support	Categorical	Grid, plate
Applied weight	Discrete numeric	0, 200, 500 g

**Table 2:** The acoustic phantom treatments measured for the design of experiments.

Phantom	Gelatin Type	Gelatin Thickness (mm)	Applied Weight (g)	Driver	Gelatin Support
1	5	2	0	Exciter	Grid
2	3	10.3	0	Exciter	Plate
3	2	2.7	200	Exciter	Grid
4	2	4	500	Speaker	Plate
5	2	3.5	500	Speaker	Plate
6	3	10.1	200	Exciter	Grid
7	3	3.6	200	Speaker	Plate
8	3	3.7	500	Exciter	Plate
9	5	9.4	500	Exciter	Grid
10	2	11.2	500	Exciter	Plate
11	3	9.7	500	Exciter	Plate
12	2	10.6	200	Speaker	Plate
13	3	6.5	500	Speaker	Plate
14	3	3.4	500	Speaker	Grid
15	5	4	0	Exciter	Grid
16	5	9.3	500	Speaker	Plate
17	3	6.8	0	Exciter	Grid
18	2	4.8	0	Speaker	Grid
19	3	5.2	500	Exciter	Grid
20	5	5.5	200	Exciter	Plate
21	2	5.8	0	Exciter	Plate
22	3	3.1	0	Exciter	Plate
23	5	4.2	500	Speaker	Grid
24	2	12.1	500	Speaker	Grid
25	3	3	0	Speaker	Grid
26	5	5.4	200	Speaker	Grid
27	3	10.2	500	Speaker	Grid
28	2	6.8	0	Speaker	Plate
29	2	5.7	500	Exciter	Grid
30	5	4	0	Speaker	Plate
31	5	9.7	0	Speaker	Grid
32	2	10.6	0	Exciter	Grid
33	5	10	0	Exciter	Plate
34	5	3.2	500	Exciter	Plate
35	5	3.3	0	Speaker	Plate

**Table 3:** The average correlation and RMSE measured between phantoms with a parameter changed.

Concern	Parameter Changed	Correlation	RMSE
Real Tissue	Gelatin (4.8 mm), 0 g	0.937	3.755
	Pig skin (4.7 mm), 0 g		
Over time	Original phantom	0.981	1.910
	Same phantom 45 days later		
Gelatin reforming	Original	0.986	1.565
	Reformed		
Gelatin curing time	90 min curing	0.998	0.670
	300 min curing		
New gelatin layers	Orignal	0.977	2.067
	New gelatin 1		
	Orignal	0.975	2.203
	New gelatin 2		
	Orignal	0.979	2.023
	New gelatin 3		
Added weight	0 g	0.980	1.954
	50 g		
	0 g	0.963	2.660
	200 g		
	0 g	0.911	4.122
	500 g		
	0 g	0.951	2.989
	Two, 200 g weights		
	Original	0.988	1.594
	After 2 days with 500 g weight		
Excitation volume	Original	0.992	1.212
	Low		
	Original	0.993	1.179
	High		

## Data Availability

Not applicable.

## Supplementary Materials

The following supporting information can be downloaded at: https://www.mdpi.com/article/10.3390/s22239086/s1, phantom construction protocol.

## Abbreviations

The following abbreviations are used in this manuscript:

DOE	Design of experiments
REW	Room EQ wizard software
RMSE	Root mean square error
SNR	Signal-to-noise ratio
