# Supplementary material for: Optimized Acoustic Phantom Design for Characterizing Body Sound Sensors
Source: Sensors (Basel). 2022 Nov 23;22(23):9086. doi: 10.3390/s22239086 (PMC9735779; doi:10.3390/s22239086)
Supplement: Supplementary file 1 [file sensors-22-09086-s001.zip › sensors-2024978-supplementary.pdf]

Supplemental Information

S1 Acoustic phantom construction

Materials and method to construct phantom for testing acoustic sensor on gelatin layer that mimics the mechanical properties of skin. The phantom is constructed with three variations in its design: (1) a loudspeaker or sound exciter to generate sound, (2) a solid or gridded gelatin support layer, and (3) various types and thicknesses of gelatins. Weight is also added to the center of the gelatin surface of several phantoms to determine its effect on the measured properties. To construct the phantom, follow the figures that are listed sequentially in the *Method* section. The links are functioning as of October 2022.

S1.1 Materials

- Polypropylene box - 2020 Target Brands - 3.25 in x 6 in x 9 in. Available here.
- Polypropylene stackable tray - 2020 Target Brands - 9 in x 6 in x 3/4 in.
- Acoustic tape.
- Magnetic tape - 0.5 inches and 1 inch.
- Acoustic foam.
- Pick pluck foam. Available here.
- Loudspeaker - HiWave BMR12 Compact 2” Full-Range square speaker 12 W 8 Ohm. Available here.
- Sound exciter - Dayton audio DAEX25. Available here.
- Larger plastic box.
- Gelatin. Various types available here. Gelatins 2, 3, and 5 are used in these experiments.
- Reflective tape. Available here.
- Audio amplifier - MAX9744. Available here.
- Slotted calibrated weights - 200 and 500 g. Available here.

S1.2 Method

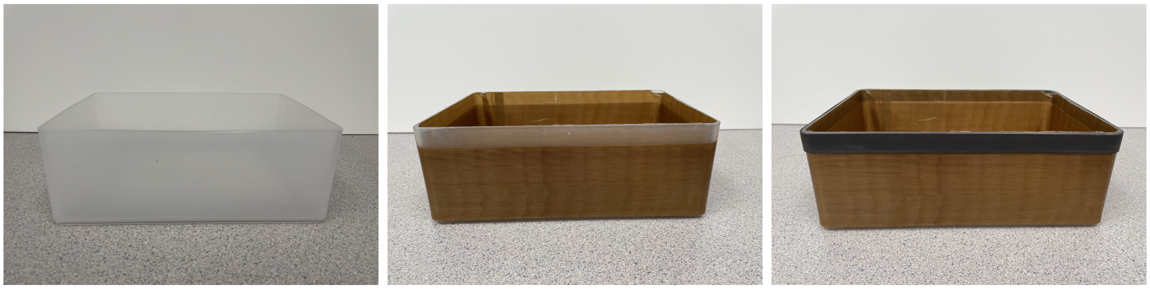

(a) (b) (c)

Figure S1: Cover polypropylene box with acoustic tape to increase sound attenuation. Add magnetic tape (0.5 inch) around the outer, top edge of the box. This will be used to hold the lid in place in subsequent steps.

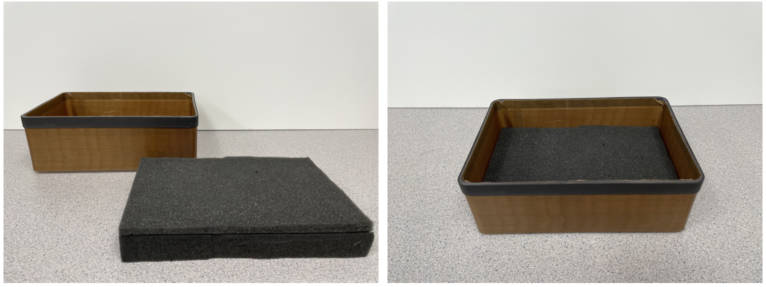

(a) (b)

Figure S2: Cut two layers of interlocking acoustic foam to the size of the box and place inside.

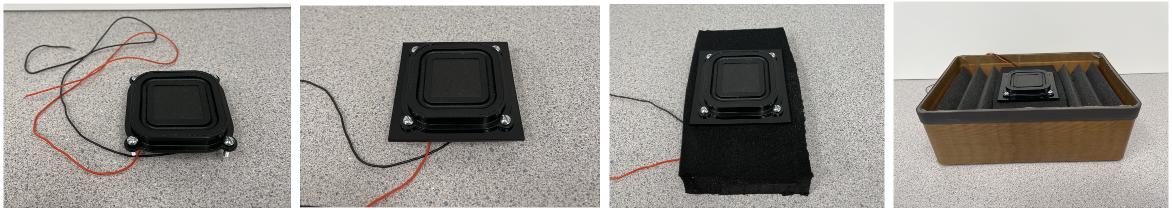

(a) (b) (c) (d)

Figure S3: **(Loudspeaker design)** Attach loudspeaker to a laser cut acrylic holder and place in pick pluck foam. Place the foam with the speaker at the center of the box and surround with two, equally sized pieces of acoustic foam. **(Sound exciter design)** Place another piece of acoustic foam on top of the first two layers.

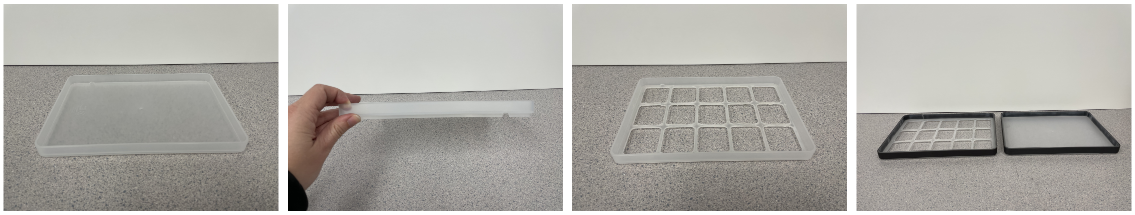

(a) (b) (c) (d)

Figure S4: Cut small notch in polypropylene tray to allow speaker wires to pass through the box and lid. Put magnetic tape around outside of lid. **(Gridded lid)** Cut 15 squares (approximately 4 cm x 4 cm) in lid using laser cutter.

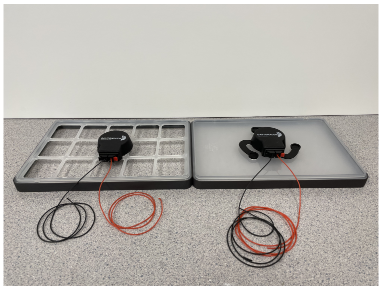

(a)

Figure S5: **(Sound exciter)** Adhere sound exciter to either grid or plate lid using super glue. For grid lid, remove legs from sound exciter.

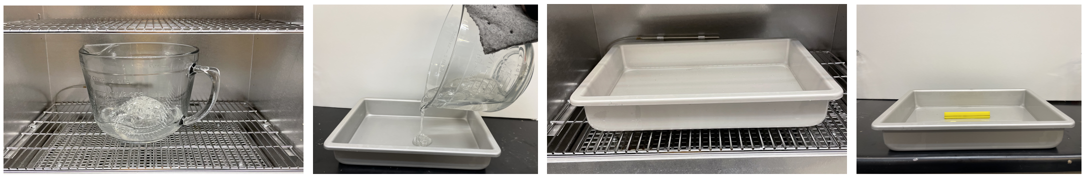

(a) (b) (c) (d)

Figure S6: Melt gelatin in the oven around 100°C, pour into baking sheet, and place back in the oven for a few minutes to create smooth surface. Make sure the approximate thickness is correct. If necessary, use a heat gun to help remove residual bubbles from the surface.

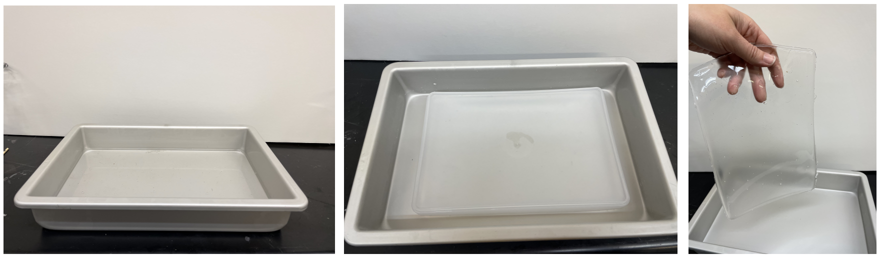

(a) (b) (c)

Figure S7: Allow gelatin to cool. Use razor blade to cut pattern out of gelatin that is approximately the same size as the lid.

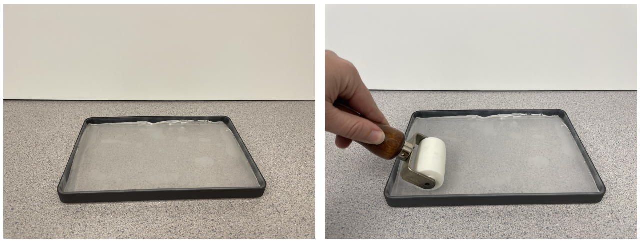

(a) (b)

Figure S8: Place gelatin in lid and roll to make sure makes it has good contact with lid. If large gaps are present between gelatin and lid, fill in with melted gelatin. Gelatin 5 is fragile, so handle carefully.

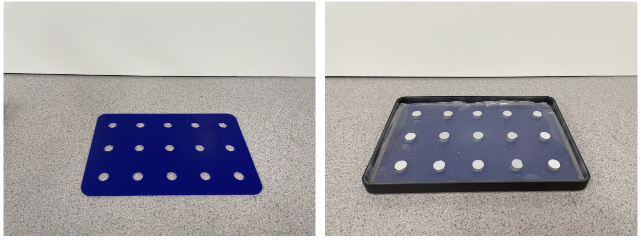

(a) (b)

Figure S9: Use acrylic pattern to place reflective tape at 15 positions across the gelatin surface. This is where the frequency response will be measured.

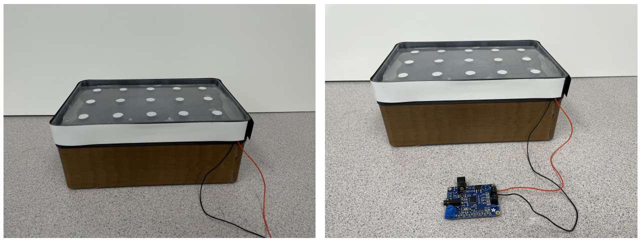

(a) (b)

Figure S10: Place the lid on the box with the wires protruding through the hole cut into the lid. Use wider magnetic tape (1 inch) to make sure no vibration occurs between the box and lid. Connect speaker wires to amplifier.

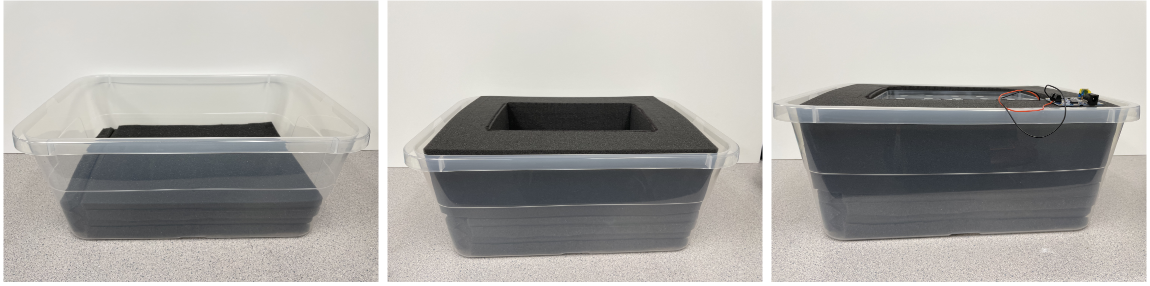

(a) (b) (c)

Figure S11: Place phantom inside larger box and surround with foam to minimize sound emanating from anywhere except the gelatin layer.
